# Hypoxic exosomes orchestrate tumorigenesis: molecular mechanisms and therapeutic implications

**DOI:** 10.1186/s12967-020-02662-9

**Published:** 2020-12-10

**Authors:** Reza Jafari, Reza Rahbarghazi, Mahdi Ahmadi, Mehdi Hassanpour, Jafar Rezaie

**Affiliations:** 1 Solid Tumor Research Center, Cellular and Molecular Medicine Research Institute, Urmia University of Medical Sciences, Shafa St, Ershad Blvd, P.O. BoX: 1138, 57147 Urmia, Iran; 2 Department of Immunology and Genetics, School of Medicine, Urmia University of Medical Sciences, Urmia, Iran; 3 Stem Cell Research Center, Tabriz University of Medical Sciences, Tabriz, Iran; 4 Department of Applied Cell Sciences, Faculty of Advanced Medical Sciences, Tabriz University of Medical Sciences, Tabriz, Iran; 5 Tuberculosis and Lung Diseases Research Center, Tabriz University of Medical Sciences, Tabriz, Iran; 6 Department of Clinical Biochemistry and Laboratory Medicine, Tabriz University of Medical Sciences, Tabriz, Iran; 7 Stem Cell and Regenerative Medicine Institute, Tabriz University of Medical Sciences, Tabriz, Iran

**Keywords:** Hypoxia, Exosomes, HIF, Tumor microenvironment, Tumorigenesis

## Abstract

The solid tumor microenvironment possesses a hypoxic condition, which promotes aggressiveness and resistance to therapies. Hypoxic tumor cells undergo broadly metabolic and molecular adaptations and communicate with surrounding cells to provide conditions promising for their homeostasis and metastasis. Extracellular vesicles such as exosomes originating from the endosomal pathway carry different types of biomolecules such as nucleic acids, proteins, and lipids; participate in cell-to-cell communication. The exposure of cancer cells to hypoxic conditions, not only, increases exosomes biogenesis and secretion but also alters exosomes cargo. Under the hypoxic condition, different signaling pathways such as HIFs, Rab-GTPases, NF-κB, and tetraspanin are involved in the exosomes biogenesis. Hypoxic tumor cells release exosomes that induce tumorigenesis through promoting metastasis, angiogenesis, and modulating immune responses. Exosomes from hypoxic tumor cells hold great potential for clinical application and cancer diagnosis. Besides, targeting the biogenesis of these exosomes may be a therapeutic opportunity for reducing tumorigenesis. Exosomes can serve as a drug delivery system transferring therapeutic compounds to cancer cells. Understanding the detailed mechanisms involved in biogenesis and functions of exosomes under hypoxic conditions may help to develop effective therapies against cancer.

## Background

Hypoxia, a condition of insufficient oxygen, is a common feature of numerous solid tumors, related to tumor development, therapy resistance, and mortality [1]. Tumor cells in the microenvironment encounter low oxygen levels because of insufficient oxygen flow and physiological anomalies in tumor vessels, resulting in normoxic, hypoxic, and also necrotic regions [2]. In most solid tumors, the average concentration of oxygen is near 10 mmHg, whereas in the other tissues it reaches 40 and 60 mmHg [3]. Tumor cells juxtaposed to the normal vessels are functional, whereas cells situated around 150 μm from the vasculature bed may undergo necrosis and atresia [4], however, cells are located between these two cell populations habituate to an insufficient oxygen environment, hypoxia. Generally, cells located in a diameter of approximately 1 mm undergo genetic, molecular, and metabolic changes. These cells could reorganize the microenvironment appropriate for growth, metastasis, and therapy resistance [5]. In this situation, by the production of pro-angiogenic factors, tumor cells can induce angiogenesis for supplying nutrients and oxygen and removing waste. Besides, increased angiogenesis in tumor mass provides a way for tumor cells to migrate and metastasize [6].

In response to a hypoxic condition, the transcription profile of tumor cells such as hypoxia-inducible factors (HIFs), the key factor involved in regulating hypoxic condition, are altered [7]. HIFs, the dimeric proteins, are composed of a constitutive subunit named HIF-1β and an oxygen-regulated α-subunit (HIF-1, 2, 3α) [8]. The activity of HIF-1α is dynamic and related to the availability of oxygen. In the presence of oxygen, HIF-1 α is degraded by the proteasome system; so that, HIF-1α is hydroxylated by prolyl hydroxylases (PHD), in keeping, von Hippel-Lindau (VHL) recognizes the hydroxylated HIF-1α. This event subsequently ubiquitinates HIF-1α for degradation by the 26S proteasome [9, 10]. PHDs are inactive in a hypoxic condition and allow HIF-1α to link to the HIF-1β subunit.

According to previous studies, HIFs are active in almost solid tumors. HIFs regulate different signaling pathways, which are implicated in the cell viability, proliferation, epithelial-to-mesenchymal transition (EMT), angiogenesis, metastasis, and therapy resistance [11]. However, other pathways including mammalian target of rapamycin (mTOR), phosphatidylinositol 3-kinases (PI3K)-Akt, Wnt/β-catenin, nuclear factor-κB (NF-κB), mitogen-activated protein kinases (MAPK), and NADPH oxidase (NOX) facilitate the adaption of tumor cells to hypoxia [1, 12, 13]. Besides, HIFs have been shown to facilitate exosomes biogenesis and secretion [14]. Exosomes a subfamily of extracellular vesicles (EVs) mediate intercellular communication by carrying different biological molecules among cells. These vesicles contain various biological molecules like proteins, RNAs (coding and non-coding RNAs), DNAs, and lipids, regulating activation of different signaling pathways in recipient cells located nearby or distant tissues [15, 16]. It has been shown that exosomes derived from tumor cells participate in modulating the tumor microenvironment and promoting tumorigenesis [17, 18]. Confirmed that, hypoxia induces exosomes biogenesis and secretion and thereby promotes tumor intercellular communication, representing the key role of exosomes in hypoxic tumors [14, 19]. The majority of experiments discussed in this review used 1% O_2_ as a hypoxic model that generally called the hypoxic condition, if not, we explained the condition. In the present review, we discuss exosomes biogenesis and loading and also possible underlying mechanisms under hypoxic. Also, we describe the key roles of hypoxic in tumorigenesis and tumor-therapy.

## Tumor microenvironment

The tumor microenvironment is a dynamic environment around a tumor, containing the blood vessels, fibroblasts, immune cells, the extracellular matrix (ECM), and signaling molecules that support proliferation, growth, metastasis, and therapy resistance of tumor cells. Tumor cell proliferation, death, invasion, migration, angiogenesis, metabolic reprogramming, immune evasion, are all regulated by the complex interaction inside the tumor microenvironment. In this regard, autocrine, paracrine, and juxtacrine communication network orchestrate these biological functions. Paracrine-mediated communication plays pivotal roles in signal transduction between neighboring and distant cells [20–22].

Non-tumor cells including fibroblasts, endothelial cells (ECs), and immune cells contribute to tumor microenvironment interaction and are affected by tumor soluble factors, and their fate goes through tumor-like modifications, persistently accommodate to the tumor microenvironment and support tumor growth. In the tumor microenvironment, fibroblasts are motivated into cancer-associated fibroblasts (CAFs), these cells are the most resident stromal cells in the tumor microenvironment, producing an ECM that vary common ECM in inflexibility and arrangement properties that facilities migration and invasion of tumor cells [23]. In the tumor microenvironment, hypoxia induces tumor cells to produce angiogenic factors, which in turn affect ECs and up-regulate angiogenesis [24, 25]. In the tumor microenvironment, the resident immune cells demonstrate multiplicity and could suppress the immune responses. Also, anti-inflammatory molecules can inhibit the immune system, which is involved in the suppression of cancer cells [26, 27]. These factors and cells make the tumor microenvironment a complex system and resistant to different therapies [28, 29].

## Biogenesis of EVs

**Fig. 1:**
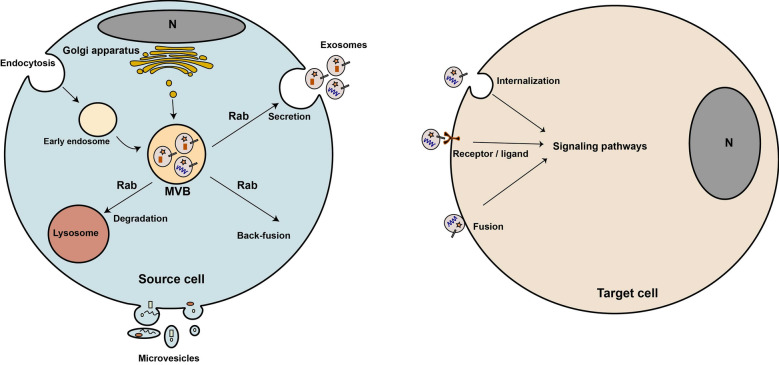
Biogenesis and secretion of exosomes. Exosomes originate from multivesicular body (MVB) and release into the extracellular matrix upon fusion of MVB with the plasma membrane. One secreted, exosomes can reach to target cells through three possible ways. Microvesicles shed directly from the plasma membrane. N: nucleus

The term EVs refers to nano-micro-sized heterogeneous vesicles released from cells via intrigue mechanisms [15]. EVs generally comprise three classes of vesicles including exosomes, ectosomes, and apoptotic bodies [30]. Exosomes are 30–150 nm vesicles that originated from multivesicular bodies (MVBs) located in the cytoplasm, and thus, secreted into the extracellular milieu following the fusion of MVBs with the plasma membrane [31] (Fig. 1). In general, MVBs have three possible fates [15, 32], including; (i) the fusion of MVBs with the plasma membrane and secretion of exosomes, (ii) fusion with lysosome and degradation of exosomes, and (iii) back-fusion with the plasma membrane and recycling the biomolecules to the plasma membrane. Upon secretion into the extracellular milieu, exosomes can deliver their cargo to target cells and affect their fate, function, and morphology [33, 34] (Fig. 1). Ectosomes are also known microvesicles (MVs), ranging from 100 to 1000 nm, shed directly from the plasma membrane [30] (Fig. 1). Similar to the exosomes loading process, proteins and lipids and of the membrane- are directed into sites of MVs budding. For example, the oligomeric cytoplasmic proteins are anchored to the plasma membrane lipids and also have a high affinity for lipid rafts, therefore these proteins can enter into MVs [35, 36]. MVs exhibit common markers such as Annexin V, Flotinin-2, CD40, CD62, and integrins.

Once EVs are secreted into the extracellular milieu, target cells can uptake these nano-sized particles. The entrance of EVs into the target cells causes phenotypic and functional changes, affecting normal and pathological states [30] (Fig. 1). According to previous studies, EVs can reach target cells by vesicle internalization, receptor-ligand interaction, and direct fusion with the target cell membrane [30, 33, 37] (Fig. 1). Vesicle internalization may comprise such different mechanisms as endocytosis, pinocytosis, and phagocytosis. In the receptor-ligand interaction way, molecules of EVs surface interact with corresponding molecules on the target cells' plasma membrane [38, 39]. In the direct fusion way, EVs membrane fuse with target cell membrane similar to conventional membrane fusion process by which EVs cargo discharged into the cytoplasm of target cells. Overall, EVs uptake is complex and possibly depends on the types of EVs and target cells, and they may be related to the downstream effects and signaling pathways mediated by EVs [37]. Furthermore, it is not clear that these ways synergically or independently are engaged by cells.

## Hypoxia promotes exosomes biogenesis

Hypoxia has been shown to induce EVs biogenesis and secretion. Wang and co-workers*,* for example, declared that exposure of breast cancer cells to hypoxia conditions results in producing more MVs via HIF/RAB22A signaling [40]. Similarly, King et al*.* [41] confirmed that incubation of different breast cancer cell lines; such as MCF-7, SKBR-3, and MDA-MB-231 cells with fair (1% O_2_) and severe (0.1% O_2_) hypoxia increased exosomes biogenesis and secretion. They also confirmed the key role of HIF in inducing exosomes biogenesis. However, the detailed mechanisms by which HIF mediates exosomes biogenesis are not fully clear. Besides, hypoxia can change the type of cargo and function of exosomes. Kucharzewska et al*.* [42]*,* for example, showed that glioma cells cultured under hypoxia condition produce exosomes containing various mRNAs and proteins such as IL-8, caveolin 1, matrix metalloproteinases (MMP), PDGFs, and lysyl oxidase, which are involved in angiogenesis. However, hypoxia can induce exosome biogenesis in non-cancerous cells. Zhang et al. [14] demonstrated that hypoxia increased exosome secretion from renal proximal tubular cells via HIF-1 signaling. Similarly, mesenchymal stem cells (MSCs) produce abundantly exosomes upon exposure to hypoxia [43, 44]. These facts show that hypoxic stress can induce biogenesis and alter exosomal cargo. The underlying molecular mechanisms regulating the exosome loading and secretion under the hypoxic condition are not fully understood yet. However, under hypoxia, few of the pivotal pathways may involve in exosomes biogenesis, which is explained in the next sections.

## HIFs signaling

In hypoxia, HIF-α protein is increased in the cytoplasm and then moves to the nucleus. In the nucleus, it links to the HIF-β subunit and another expression regulators such as the co-activators p300/CBP. Then, this complex binds to the conserved hypoxia-responsive element (HRE) for the expression of genes [45]. HIFs both directly and indirectly mediate the formation of exosomes. Previous studies showed that HIF-1α is involved in MVs and exosome biogenesis in different cell lines such as tumor and non-tumor cells [16, 20, 22]. A mediator molecule that HIF-1 regulates exosome biogenesis under hypoxia is pyruvate kinase 2 (PKM2) enzyme [46], which facilities the progression of the final step within glycolysis, the dephosphorylation of phosphoenolpyruvate to pyruvate, and it participates in ATP production within the glycolytic cycle [47]. We know that the tumor environment represents a high level of lactate as a result of glycolysis. In this regard, there is evidence that PKM2 phosphorylates synaptosome-associated protein 23 (SNAP-23), a protein associated with the synaptosome/SNARE complex, and increases secretion of exosomes [48]. Blocking of glycolysis has been confirmed to diminish the number of exosomes while increasing glycolysis with TNF-α enhanced the number of exosomes [48]. Under hypoxia conditions due to higher glycolysis, the accumulation of lactate causes the acidic condition in the extracellular environment. The acidic pH increases exosome secretion while alkaline pH decreases exosome abscission. As matter of fact, the loading of protein and RNA into exosome is affected by the pH of the environment [49, 50].

## Rab-GTPases signaling

Different Rab proteins regulate the intracellular trafficking of MVBs/exosomes [15]. Rab-GTPases belong to the Ras superfamily of small GTPases, which are associated with the vesicles and the inner side of the plasma membrane, participating in intracellular trafficking of vesicles [51]. The Rab proteins have two distinct forms as an active form (GTP-bound) and an inactive form (GDP-bound. Rab- GTP-bound form is active and associated with effector proteins, and mediate formation and movement of vesicles incorporation with actin and tubulin [52]. Dorayappan et al*.* [53] declared that hypoxia could up-regulate STAT3 in ovarian cancer cells. STAT3 regulates the expression of Rab27a and Rab7 molecules; thus increases exosomes biogenesis. Panigrahi et al*.* [54] showed that Rab5 protein was accumulated around the perinuclear region of prostate cancer cells upon exposure to the hypoxic condition. RAb5 mediates the movement of the endocytosis vesicles from the plasma membrane to early endosomes and also the fusion of early endosomes with each other, proposing a key role of Rab5 in MVBs development and, subsequently promoting exosome biogenesis [54]. Hypoxia can increase the expression of the RHO-associated protein kinase (ROCK) gene, a pivotal enzyme in remodeling actin and cytoskeleton components [55]. ROCK contributes to MVs biogenesis [56], however, it was demonstrated that RhoA can drive the ROCK signaling, which subsequently leads to an induces in exosome biogenesis in various tumor cells [56].

## NF-κB signaling

The exact effect of hypoxia on NF-κB expression/induction is not clear yet. However, there is evidence that PHDs regulate the expression of NF-κB under a hypoxic condition. The direct effect of NF-κB signaling on exosome biogenesis is not fully discovered so far. Hypoxia can induce the NF-κB signaling pathway, which in turn impacts the exosome cargo loading process. For example, Yang et al*.* [57] declared that suppression of NF-κB could alter the content of the redox modulating enzymes in exosomes derived from sera of NF-κB knockout mice. Inhibition of NF-κB by aspirin leads to a significant decrease in exosomes biogenesis and secretion [57]. Other molecules may induce exosome biogenesis. Formation of the reactive oxygen species under hypoxic conditions can induce exosome release. Hedlund et al*.* [58]*,* for example, indicated that under oxidative stress (H_2_O_2_ treatment) Jurkat cells and Raji cells abundantly secrete exosomes. Besides, the authors showed that the exosomes' secretion rate of both the cell lines was increased upon the incubation of cells with thermal stress conditions.

## Tetraspanin signaling

Tetraspanin proteins including CD9, CD63, CD37, CD82, and CD81, which are widely used as markers for exosomes, have been shown to facilitate exosome formation during the hypoxic condition. A hypoxia-regulator factor is located in the upstream region of the mouse CD82 gene that is up-regulated by HIF under hypoxia [59]. By overexpressing CD9 and CD82 molecules in HEK 293T cells, Chairoungdua and co-workers confirmed that exosomes production significantly increase [60]. Other key molecules including NOX2 and PI3K/mTOR axis are enriched in hypoxic cells-derived exosomes [61, 62], however, the exact role of them in exosomes biogenesis remains unclear.

## Hypoxia can change exosomes cargo

Hypoxia can alter exosomes cargo. For example, by in vitro and in vivo, Jung et al*.* [63] showed that exosomes derived from hypoxic mouse breast cancer cells contain abundantly miR-210 that is involved in regulating vascular remodeling-related genes, including PTP1B and Ephrin A3. Similarly, Ding et al*.* [64] reported that inducing hypoxic stress (1% O_2_) on SKOV3 ovarian cancer cells led to an increase in the exosomal miR-210 level. The authors concluded that miR-210 increased SKOV3 ovarian cancer cells' mobility and progression. HIF-1α interacts with HRE, which is located on the proximal end of miR-210 and directs it into exosomes [65]. Hypoxic colorectal cancer (CRC) cells release exosomes containing a higher level of Wnt4 that increase the normoxic (21% O_2_) CRC cell migration behavior and invasion rate via HIF-1α signaling [66]. Furthermore, exosomal Wnt4 increases β-catenin transposition to the nucleus in normoxic CRC cells. The activation of the β-catenin signaling pathway plays a pivotal role in the motility and invasion of normoxic CRC cells. In another study, human pancreatic cancer cells such as PCA LNCaP and PC3 were cultured under hypoxic (1% O_2_) or normoxic (21% O_2_) conditions, and their exosomes were isolated from conditioned media. Immunoblotting analysis showed that the protein levels of CD63, CD81, HSP90, HSP70, and Annexin II were increased in hypoxic exosomes as compared to normoxic ones [67]. Exosomes released from hypoxic U87MG glioblastoma cells contain an elevated level of some proteins like thrombospondin-1 (TSP1), VEGF, and protein-lysine 6-oxidase (LOX) that promote growth, metastasis, and angiogenesis of tumor [68]. Using hypoxia-resistant multiple myeloma (HR-MM) cells, Umezu and colleagues team found that these cells abundantly produce exosomes with distinct cargo following incubation with the chronic hypoxic condition. They showed that these exosomes are enriched with miR-135b as compared to those of normoxic cells [69]. The impact of hypoxia on exosomes cargo derived from different cancer cells has been studied, and we summarized a list of the altered cargo of exosomes under hypoxic condition in Table 1.

**Table 1:** Altered cargo of exosomes under hypoxic condition

Cancer	Breast	Ovarian	Pancreas	Myeloma	Gliablastoma	Prostate	Colorectal
Cell line	MCF7, SKBR3, and MDA-MB 231/ 4T1	SKOV3, HO-8910	BxPC-3 and AsPC-1	RPMI8226, KMS-11, and U266	U87MG	LNCaP and PC3 cells LNCaP, 22Rv1, PC3, and PWR-1E	HT29 and HCT116 CRC patients
Hypoxic condition	1% O_2_ and 0.1% O_2_	1% O_2_	1% O_2_	1% O_2_	1% O_2_	1% O_2_	1% O_2_
Cargo (Increased)	miR-210 [41, 63]	miR-21–3p, miR-125b-5p, and miR-181d-5p [132]	miR-21 [133]	miR-135b [134]	HIF mRNA, LOX, TSP1, VEGF, disintegrin, MMP, and ADAMTS1 [68] MMPs, IL-8, PDGFs, caveolin 1, and lysyl oxidase [107]	CD63, CD81, HSP90, HSP70, and Annexin II [67] Triglycerides, omega-6 fatty acids, linoleic acid, arachidonic acid, palmitic acids, stearic acids, and linoleic acids [135] lactic acid [136]	Wnt4 [137] miR-210 [108]

## Role of hypoxic exosomes in tumor cell metastasis

Metastasis, relocation of tumor cells from the origin site to a secondary location, is an essential factor for cancer growth [70]. Tumor cells release exosomes with aggressive properties that can promote tumorigenesis via increasing metastasis upon reach to recipient cells [71]. The induction of epithelial-mesenchymal transition (EMT) in tumor cells is a critical event in the initiation of metastasis [72].

During the EMT process, the tumor cells reorganize their cytoskeleton and acquire a mesenchymal-like phenotype, which enables them for migration and invasion to the new locations [73]. In this scenario, the E-cadherin molecules are down-regulated, which is pivotal for the loss of epithelial phenotype [74]. Thus, loss of E-cadherin is associated with the reduction of cell-to-cell contact, the disruption of catenin/ E-cadherin interaction, abnormal β-catenin signaling, and finally cytoskeleton reorganization. Collectively, these events cause losing epithelial shape and acquiring a migratory phenotype in tumor cells [75].

A growing body of evidence showed that hypoxic tumor cells secrete exosomes promoting the migration ability and invasion rate of different tumor cells. For example, it was reported that exosomes collected from conditioned media of hypoxic prostate cancer cell contain several proteins that down-regulated adherents molecules in naïve LNCaP and PC3 prostate cancer cells, which, in turn increased the migration and invasiveness of cells, thus prompting metastasis in vitro [67]. The authors also showed that these exosomes down-regulated E-cadherin coincided with the up-regulation of nuclear and cytoplasmic β-catenin in prostate cancer cells, indicating an increase in the invasiveness, and stemness of prostate cancer cells. Xue et al*.* [76] found that hypoxia increased the loading of lncRNA-UCA1 into exosomes of bladder cancer cells. These exosomes promoted the growth and migration of bladder tumor cells both in vitro and animal models. Besides, they showed that the expression levels of lncRNA-UCA1 in blood exosomes of patients with bladder cancers were higher in comparison with the healthy ones. Similarly, Li and co-workers showed that exosomes isolated from hypoxic oral squamous cell carcinoma could induce metastasis in normoxic cells. Further scrutiny revealed that these exosomes abundantly contain miR-21, which was responsible for tumorigenesis via regulating the expression of E-cadherin, snail, and vimentin in target cells [77]. Besides, exosomes derived from hypoxic-treated hepatocellular cancer cells are enriched with linc-RoR that promote migration and metastasis of tumor cells via a miR-145/HIF-1α signaling pathway in vitro [78]. LMP1-positive exosomes derived from nasopharyngeal carcinoma cells can increase metastasis in recipient nasopharyngeal cells via transporting HIF1α and inducing EMT phenotype [79]. The authors demonstrated that HIF1α altered the expression of E- and N-cadherins which is associated with EMT and led to increased migration of cells in vitro. The results obtained from Wang et al*.* [80] study showed that hypoxic exosomes derived from metastatic small cell lung cancer cells (NCI-H1688) induced migration of tumor cells through TGF-β and IL-10 in vitro. Several experiments have been carried out to investigate the effect of hypoxic exosomes on tumor invasiveness and metastasis (Table 2).

**Table 2:** Roles of exosomes derived from hypoxic tumor cells in cancer

Cancer	Cell line	Hypoxic condition	Exosome cargo	Mechanism	Function
Colorectal	HT29 and HCT116	1% O_2_	Wnt4	Up-regulates β-catenin nuclear translocation in endothelial cells	Increase angiogenesis [137]
Glioblastoma multiforme	U87MG	1% O_2_	TF	induces TF/VIIa-mediated PAR-2 activation in endothelial cells	Increase angiogenesis [138]
Renal	Caki-1, KMRC-1, OSRC-2 and 786-O cells	1% O_2_	CA9	Increases its target MMP2	Increase angiogenesis [139]
Prostate	PCA LNCaP and PC3 cells	1% O_2_	Proteins	ND	Increase invasion and migration [67]
Nasopharyngeal	CNE1 and CNE2 TW03, C666 and CNE2 and the NP69	1% O_2_ 1% O_2_	MMP13 HIF-1α miR-24-3p	ND Induces EMT ND	Increase invasion and migration [140] Modulate immune response [141]
Lung and leukemia	IGR-Heu lung carcinoma cell line / K562, NK92, and NKD cell lines	0.1% O_2_	TGF-β1 and miR-23a	TGF-β1 suppresses NKG2D, and miR-23a directly inhibits CD107a	Modulate immune response [142]
Bladder	5637 cells	1% O_2_	linc-UCA1	Induces EMT	Increase invasion and migration [143]
Hepatocellular	HepG2, Hep3B, PLC-PRF5 and Huh-7	21% O_2_	linc-RoR	ND	Increase invasion and migration [78]
Breast cancer	4T1	0.1% O_2_	miR-210	Suppresses the expression of Ephrin A3 and PTP1B	Increase angiogenesis [63]
ovarian	SKOV3	1% O_2_	miR-940	Induces M2-type macrophages exhibiting CD163 and CD206 markers	Modulate immune response [89]
lung	A549, H1299, and HCC827	1% O_2_	miR-494 miR-23a	Suppresses PTEN and activates Akt/eNOS pathway in endothelial cells Down-regulating PHD1, PHD2 and ZO-1	Increase angiogenesis [93] Increase angiogenesis [92]
Leukemia	K562	1% O_2_	miR-210	Inhibits the expression of Ephrin A3	Increase angiogenesis [101]
Oral squamous cell	SCC-9 and CAL-27	1% O_2_	miR-21	Induces EMT	Promote invasion and migration [144]
Multiple myeloma	RPMI8226, KMS-11, and U266	1% O_2_	miR-135b	Down-regulates FIH-1	Increase angiogenesis [134]

## Role of hypoxic exosomes in modulating immune responses

**Fig. 2:**
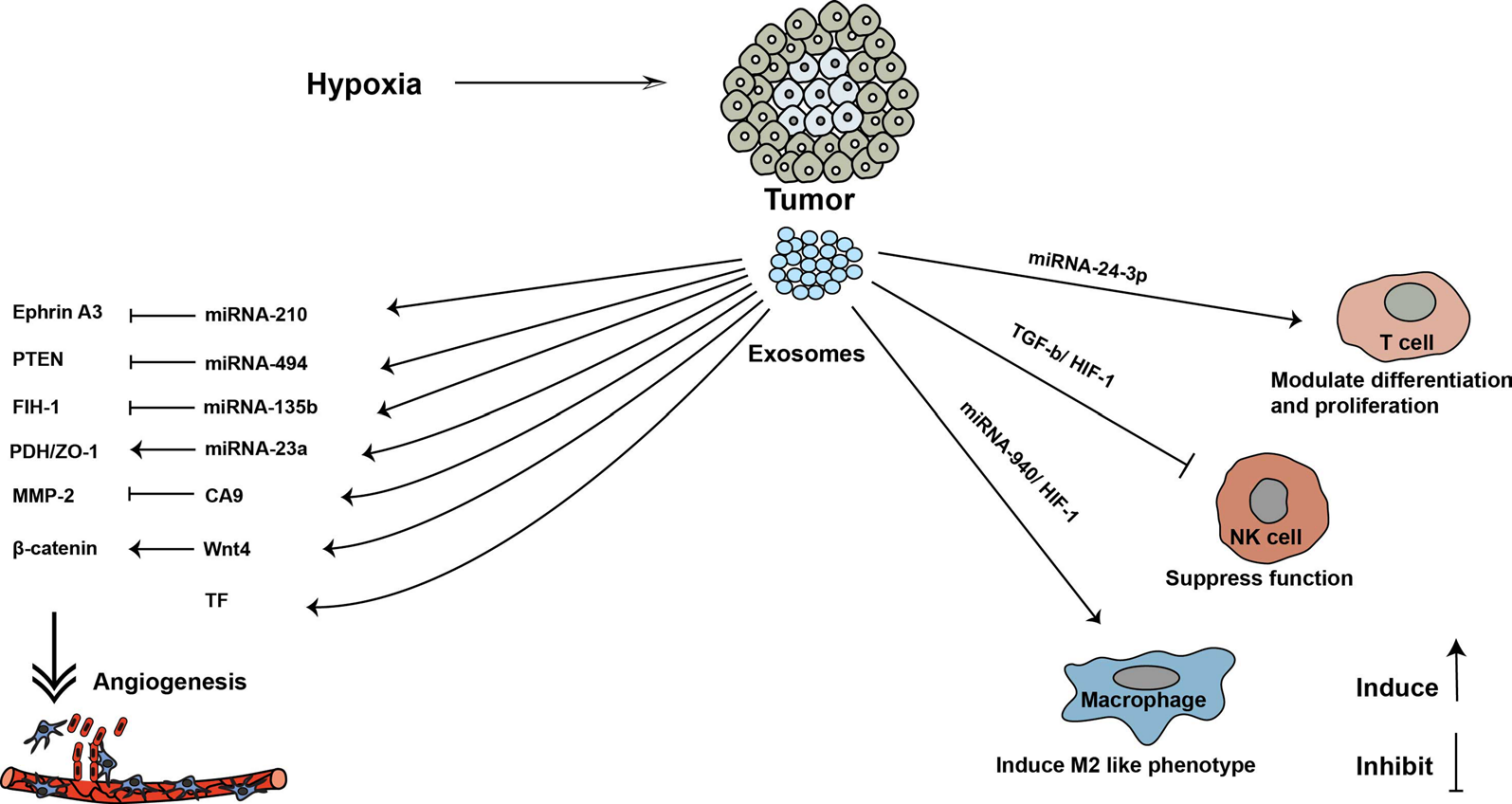
Effect of exosomes derived from hypoxic tumor cells on immune cells and angiogenesis

Modulating immune responses is vital for the invasion and formation of a new niche for the progress of metastasis [81]. Exosomes from different tumor cells have been shown to modulate the immune system via different ways such as suppression of NK cells activity, inducing T-cell apoptosis, down-regulating IFN-γ-inducible class II expression of macrophages, and regulating the differentiation of monocytes into the myeloid-derived suppressor cells (MSDCs), which collectively leads to immunosuppressive function and the cancer development [82, 83] (Fig. 2). Exosomes from hypoxic nasopharyngeal carcinoma (NPC) cells contain miR-24-3p that inhibit T-cell proliferation, Th17, and Th1 differentiation; and promote the differentiation of regulatory T-cells (Tregs) [84]. Exosomes derived from breast cancer cells directly inhibited the T-cell survival and the NK cell function, consequently, the immune response was inhibited against tumor cells in pre-metastatic organs [85]. Exosomes from lung cancer cells transfer miRs that inhibit the genes related to the Toll-like receptor (TLR) family in macrophages. This event induces the production of proinflammatory cytokines that supports enhanced tumor dissemination [86]. Tumor-derived exosomes from plasma of head and neck squamous cell carcinoma patients contain CD73 and CD39 molecules, which participate in the production of adenosine from ATP. Adenosine is involved in suppressing the activity of activated B cells by converting the motivated B-cells into regulatory B-cells [87]. Also, Liu et al*.* [88] demonstrated that exosomes from mouse breast and melanoma tumor cells induced the differentiation of myeloid precursor cells towards MSDCs. This phenomenon promotes tumor exosome-mediated expansions of MDSCs and tumor metastasis. Incubation of epithelial ovarian cancer with the hypoxic condition may promote the loading of miR-940 into exosomes. These exosomes induce macrophage polarization by up-regulating the expression of the M2-type markers like CD206 and CD163. These findings demonstrated that exosomes transferred miR-940 to macrophages and induced macrophages to an M2-like phenotype, which is favorable for tumor progression [89]. Collectively, tumor-derived exosomes negatively modify immune cell function and participate in tumor growth.

## Role of hypoxic exosomes in tumor angiogenesis

Angiogenesis, the progress of new blood vessels from the pre-existing vessels, is a complex process and a critical factor that promotes the growth and metastasis of several solid tumors [90]. Hypoxia is frequently seen in the tumor microenvironment and up-regulates angiogenesis [91]. This process is highly regulated by various ligands, receptors, and several signaling pathways. Angiogenesis switch on/off is related to the balance between anti and pro-angiogenic factors. A growing body of evidence has highlighted the key role of the miRs cargo of hypoxic exosomes in tumor angiogenesis. A study by Umezu and co-workers demonstrated that miR-135b is abundantly present in hypoxia-resistant multiple myeloma (HR-MM) cells that enhance angiogenesis in human umbilical vein endothelial cells (HUVECs) through targeting HIF-1 in vitro [69]. Hypoxic lung cancer cells secrete exosomes enriched with miR-23a that facilitate the angiogenesis in HUVECs by down-regulating ZO-1 and prolyl hydroxylase proteins. These proteins are involved in tight junction [92]. Hypoxia increased miR-494 molecules in exosomes of non-small cell lung cancer (NSCLC) cells through the HIF-1α-mediated mechanism. These exosomes down-regulated PTEN and activated Akt/eNOS axis in HUVECs, and therefore promoted angiogenesis [93]. Recently, an analysis of exosomes from hypoxic human breast cancer cell line (MDA-MB-231 cells) showed that these exosomes contain many types of angiogenic mRNAs that can promote angiogenesis in HUVECs. Co-culturing of these exosomes with HUVECs could deliver exosomal VEGF-A mRNAs from tumor cells to HUVECs and were translated into protein in HUVECs, therefore induced VEGFR2-dependent angiogenesis [94]. Mao et al*.* [95] reported that exosomes released from hypoxic esophageal squamous cell carcinoma increased growth, migration, invasion, and tubulogenesis of HUVECs both in vitro and in vivo as compared to exosomes derived from normoxic cells. Under hypoxic conditions, pancreatic tumor cells release exosomes enriched with noncoding RNA UCA1 molecules that promote angiogenesis via miR-96-5p/AMOTL2/ERK1/2 axis both in vitro and in vivo [96]. Exosomes derived from hypoxic glioblastoma cells have been shown to transfer miR-182-5p that can directly inhibit miR-182-5p targets Kruppel-like factor 2 and 4, resulting in the up-regulation of VEGFR, thus increasing tumor angiogenesis. Furthermore, exosomal miR-182-5p can target tight junction-related proteins including occludin, ZO-1, and claudin-5 in ECs which result in promoting migration and metastasis [97]. A study by Matsuura et al*.* [98] demonstrated that exosomes isolated from human liver cancer cell lines (PLC/PRF/5 and HuH7) increased the angiogenic activity of HUVECs. They showed that these exosomes were enriched with miR-155 responsible for inducing angiogenesis in HUVECs. Exosomes derived from hypoxic papillary thyroid tumor cells including KTC-1 and BCPAP cells promoted the angiogenesis in HUVECs compared with those of normal thyroid follicular cell lines (Nthy-ori-3-1), normoxic BCPAP cells both in vitro and in vivo*.* In keeping, it was demonstrated that hypoxic exosomes contain high levels of miR-21, which targeted COL4A1 and TGFBI andinduced tubulogenesis and angiogenesis [99]. More recently, Mo et al. [100] found that exosomes derived from hypoxic A549 lung cancer cells contain angiopoietin-like 4 protein that induces angiogenesis in HUVECs. Also, exosomal miR-210 released from hypoxic leukemia cells induced tubulogenesis in ECs [101]. These data show exosomes from hypoxic tumor cells can promote angiogenesis, thus these exosomes may serve as a novel target for cancer treatment.

## Diagnostic application of hypoxic exosomes

Early diagnosis of cancer is the hallmark of cancer therapy that improves the survival rate and quality of a patient’s life [102]. As exosomes released from tumor cells can be distributed to several bio-fluids, thus, a simple liquid-biopsy from plasma, serum, urine, and CSF is a non-invasive way for acquiring detailed information about tumor environment/status [15]. As exosomes originate directly from tumor cells, they may serve as a diagnostic tool for predicting the extent of the physiological and pathological status of tumor cells. Exosomes cargo like proteins and nucleic acids are altered upon the change in the dynamic of parental cells, suggesting a prognostic and diagnostic tool for the treatment of cancer [103]. Analyzing exosomal cargoes (miRs and proteins) gives a chance for scientists to predict the status of a pathological condition like tumor progression. Therefore, as under hypoxia condition tumor cells release more exosomes with distinct cargoes; they may be potentially used as a biomarker for hypoxia tumors. Exosomal biomarker represents superiority against other approaches evaluating hypoxia. Currently, some of the approaches have clinical challenges in estimating hypoxia. For instance, pimonidazole and immunohistochemistry techniques are invasive and necessitate surgical elimination of tumors. Consequently, the application of tumor-derived exosomes from bio-fluids to obtain evidence of hypoxia standing in various cancers could be noteworthy. In this regard, exosomal miRs and protein obtained from bio-fluids have biomarker potential for the diagnosis of different cancers [104, 105]. For example, Matsumura et al*.* [106] reported that expression of miR-19a in exosomes isolated from the serum of CRC patients was up-regulated, which could be considered as a relapse biomarker of CRC. In the case of hypoxic tumors, confirmed that HIF-1 mRNA molecules are enriched within tumor-derived exosomes that are commonly considered as a typical biomarker for diagnosing cancer development as well as therapy consequences [79]. A study conducted by Kucharzewska et al*.* [107] demonstrated that exosomes obtained from both Glioblastoma multiform (GBM) cells culture medium and isolated from the GBM patients plasma abundantly contain hypoxia-regulated proteins and mRNAs including PDGFs, Caveolin 1, IL- 8, MMPs, and LOX. The authors conclude that the mRNA and proteome content of these exosomes reflect the hypoxic status of cancer and have biomarker potential for GBM. Also, miRs and metabolites cargo of hypoxic exosome would be useful as a biomarker for diagnosis and prognosis of different cancers such as prostate, colorectal, and pancreatic cancers [108–110]. For example, the expression pattern of exosomal miR-210 from the serum of CRC patients may function as a promising non-invasive biomarker for the diagnosis and prognosis of CRC [108]. During the hypoxic condition, miR-210 is the most extensively and consistently up-regulated miR that commonly shows tumorigenesis properties in different tumors [111]. Similar to hypoxic exosomes from prostate tumor cells (PCa and LNCaP), exosomes obtained from the serum of PCa patients contain a high level of miR-885 and miR-521 [109]. Besides, proteins cargo (VLA-4, TYRP2, HSP90, and HSP70) of exosomes derived from the plasma of melanoma patients are significantly increased in comparison with healthy persons [112]. VLA-4 and TYRP2 are up-regulated under the hypoxic condition and their high expression in exosomes correlates with stage 3 melanoma [113, 114]. Previous studies have shown that HSP90 and HSP70 are hypoxic related proteins and play roles in hypoxic condition [115, 116]. In this regard, exosomal proteins have diagnostic and prognostic value for the melanoma tumor development and hypoxic status. Therefore, hypoxic exosomes may be a useful tool for predicting the hypoxic status of solid tumors, however, it seems that this evidence is not sufficient and further scrutiny is essential to examine and confirm the potential application of hypoxic exosomes to quantify the degree of hypoxia to detect stages of tumor development.

## Possible therapeutic application of exosomes

Exosomes can reach target cells and alter the function, fate, and morphology through different signaling pathways. As mentioned, tumor cells under hypoxic conditions produce more exosomes, promoting tumorigenesis. Thus, it seems that targeting exosomes formation and secretion particularly from the hypoxic tumor may provide us with a tool that reduces tumorigenesis. Recent findings have shown that it is possible to inhibit the exosomes biogenesis and secretion from different cells. For example, Manumycin A and GW4869 have been shown to inhibit exosome biogenesis and release from cells [117]. Datta et al*.* [118] reported that Manumycin A inhibited exosomes biogenesis and secretion from aggressive prostate cells mainly by suppression of Ras/Raf/ERK1/2 signaling and hnRNP H1. They concluded that Manumycin A is a potential drug candidate to inhibit exosome biogenesis and secretion. Suppression of Rab27a, a protein involved in exosomes secretion, has been shown to inhibit exosome-dependent and -independent tumor cells growth [119]. Inhibition of Rab proteins involved in intracellular trafficking of exosomes/MVB may inhibit exosomes biogenesis and release, thus they may be a target to inhibit exosome biogenesis. For example, Rab5a has been involved in the early step of exosomes biogenesis, while Rab11, Rab27a, and Rab35 regulate the MVBs-plasma membrane fusion and exosomes secretion. Moreover, inhibition of sphingomyelinase, an enzyme catalyzes the formation of ceramide from sphingomyelin, may lessen exosomes biogenesis and loading, thus prevents tumor growth [120]. In human prostate cancer (PC3) cells, it was shown that Imipramine profoundly inhibited the biogenesis of both microvesicles and exosomes [121]. A fascinating approach has been proposed by Marleau and colleagues based on the effective elimination of blood exosomes of breast cancer patients by extracorporeal hemofiltration associated with affinity agents like exosome-trapping antibodies and lectins. This approach was proposed to trap particles < 200 nm from the whole circulatory system [122]. Considering the existence of numerous experiments on exosomes inhibition, there are challenges regarding analyzing and conclusions of findings, because as various methods are used to isolate and characterize exosomes. Moreover, some researchers did not include ISEV guidelines regarding the exosomes confirmation and validation, as exosome-based studies had been performed before the 2014 and 2018 declaration of ISEV guidelines about exosome-based studies [30, 123]. Besides, it is vital to discover the non-toxic doses of the drugs for target cells to confirm that any decrease in exosomes secretion resulting from exosomes inhibition not from cell death. The majority of these experiments were pre-clinical performed, thus, clinical trials are essential for the approval. Furthermore, the non-targeting effects of these drugs on exosomes biogenesis from normal cells remain an important concern. At least, in the field of cancer, key studies must still be necessary to investigate their effects on exosomes production from both healthy and tumor cells and to progress methods to specifically deliver drugs into tumor cells.

Another approach that exosomes can be used as a therapeutic agent is the drug delivery potential of them. According to previous studies, exosomes can be used as a drug delivery system in two ways: (I); direct loading by which therapeutic agents directly sorted into exosomes; and (II); indirect loading where source cells co-cultured with therapeutic agents or manipulated genetically to produce optional exosomes. In this regard, different approaches for producing optional exosomes have been examined, which comprise: incubating exosomes with the agents, electroporation, sonication, sensitive fusogenic peptide, and cationic lipid, liposome, and exosome-coated metal–organic nanoparticle [124, 125]. An example of the direct method, Zhuang et al. [126] encapsulated curcumin into tumor cell-derived exosomes. Then, these exosomes successfully delivered these exosomes to microglia cells through an intranasal way in a brain inflammatory model of mice. Also, the authors successfully inserted a Stat3 inhibitor into the same exosomes. They demonstrate that this method could provide a noninvasive and new therapeutic approach for the treatment of brain diseases. Besides drugs, siRNAs can also be loaded into the hydrophilic core of exosomes in the pharmaceutically active form [127, 128]. In this regard, Alvarez-Erviti et al*.* [129], for example, successfully loaded siRNAs into exosomes purified from dendritic cells and delivered those exosomes to the mouse brain.

An example of an indirect loading method, Pascucci et al*.* [130] co-cultured MSCs with paclitaxel (PTX) and then isolated exosomes from the supernatant. In keeping, they found that PTX was sorted into exosomes and these exosomes exhibited strong anti-tumor activity in vitro. Interestingly, exosomes from hypoxic MDA-MB-231 human breast cancer cells loaded with anti-cancer drug Olaparib exhibited better uptake rate when co-cultured with hypoxic cancer cells [131]. Collectively, exosomes may serve as a new avenue to overcome cancer, however, translation of pre-clinical results into the clinic needs more experiments regarding exosomes biology and bio-applications in disease models.

## Conclusion

Hypoxia increases exosome biogenesis and secretion in tumor cells. Moreover, it can alter exosomes cargo. Exosomes released from tumor cells play a pivotal role in promoting growth, metastasis, and resistance of hypoxic tumors. Furthermore, exosomes from hypoxic tumors have been suggested to be a promising non-invasive biomarker for cancer diagnosis through analyzing their components such as proteins and miRs. Inhibition of exosomes biogenesis and secretion may help to reduce tumorigenesis. Exosomes can be used as a drug delivery system for the treatment of cancer. However, despite many experiments, translation of the preclinical findings into the clinic requires additional examinations in this field. Therefore, further scrutiny is essential for a better understanding of the mechanisms behind exosome loading and production under hypoxic conditions, which could be useful in targeting exosomes biogenesis and prevent tumorigenesis.

## Data Availability

The primary data for this study is available from the authors on direct request.

## Abbreviations

ABs: Apoptotic bodies
AMPK: AMP-activated protein kinase
CAFs: Cancer-associated fibroblasts
CMA: Chaperone-mediated autophagy
CML: Chronic myeloid leukemia
d-GalN/LPS: D-galactosamine and Lipopolysaccharide
GBM: Glioblastoma multiform
ECM: Extracellular matrix
ECs: Endothelial cells
EMT: Epithelial-to-mesenchymal transition
ESCRT: Endosomal sorting complex required for transport
EVs: Extracellular vesicles
HIFs: Hypoxia-inducible factors
HRE: Hypoxia-responsive element
HR-MM: Hypoxia-resistant multiple myeloma
HUVECs: Human umbilical vein endothelial cells
ILVs: Intraluminal vesicles
ISEV: International Society for Extracellular Vesicles Protein type 2A
LOX: Protein-lysine 6-oxidase
MAPK: Mitogen-activated protein kinases
MHC: Major histocompatibility complex
MMPs: Matrix metalloproteinase
MSCs: Mesenchymal stem cells
MSDCs: Myeloid-derived suppressor cells
mTOR: Mammalian target of rapamycin
MV: Microvesicles
MVBs: Multivesicular bodies
nSMase2: Sphingomyelinase 2
NSCLC: Non-small cell lung cancer
NOX: NADPH oxidase
NPC: Nasopharyngeal carcinoma
PI3K: Phosphatidylinositol 3-kinases
PHD: Prolylhydroxylase
PKM2: Pyruvate kinase 2
PLP: Proteolipid proteins
PM: Plasma membrane
PTX: Paclitaxel
ROCK: RHO-associated protein kinase
ROS: Reactive oxygen species
SNAREs: Soluble NSF attachment protein receptors
TLR: Toll-like receptors
VHL: Von Hippel-Lindau
